# Supplementary material for: The Basic Properties of Gold Nanoparticles and their Applications in Tumor Diagnosis and Treatment
Source: Int J Mol Sci. 2020 Apr 3;21(7):2480. doi: 10.3390/ijms21072480 (PMC7178173; doi:10.3390/ijms21072480)
Supplement: Supplementary file 1 [file ijms-21-02480-s001.pdf]

Topic	Reference
**Anisotropic nanoparticles**	[1] Hao, E., G.C. Schatz, and J.T. Hupp, Synthesis and optical properties of anisotropic metal nanoparticles. J Fluoresc, 2004. 14(4): p. 331-41.
	[2] Li, N., P. Zhao, and D. Astruc, Anisotropic gold nanoparticles: synthesis, properties, applications, and toxicity. Angew Chem Int Ed Engl, 2014. 53(7): p. 1756-89.
	[3] Jones, S., et al., Recent progress on the development of anisotropic gold nanoparticles: Design strategies and growth mechanism. J Environ Sci Health C Environ Carcinog Ecotoxicol Rev, 2017. 35(1): p. 47-66.
	[4] Reguera, J., et al., Anisotropic metal nanoparticles for surface enhanced Raman scattering. Chem Soc Rev, 2017. 46(13): p. 3866-3885.
	[5] Kohout, C., C. Santi, and L. Polito, Anisotropic Gold Nanoparticles in Biomedical Applications. Int J Mol Sci, 2018. 19(11).
**Atomic number**	[6] Coulter, J.A., et al., Radiosensitising nanoparticles as novel cancer therapeutics--pipe dream or realistic prospect? Clin Oncol (R Coll Radiol), 2013. 25(10): p. 593-603.
	[7] Popescu, R.C., M.O. Fufa, and A.M. Grumezescu, Metal-based nanosystems for diagnosis. Rom J Morphol Embryol, 2015. 56(2 Suppl): p. 635-49.
	[8] Torrisi, L., Gold nanoparticles enhancing protontherapy efficiency. Recent Pat Nanotechnol, 2015. 9(1): p. 51-60.
	[9] Titus, D., E.J. Samuel, and S. Mohana Roopan, Current scenario of biomedical aspect of metal-based nanoparticles on gel dosimetry. Appl Microbiol Biotechnol, 2016. 100(11): p. 4803-16.
	[10] Azharuddin, M., et al., A repertoire of biomedical applications of noble metal nanoparticles. Chem Commun (Camb), 2019. 55(49): p. 6964-6996.
**Biocompatibility**	[11] Nie, L., et al., Applications of gold nanoparticles in optical biosensors. J Biomed Nanotechnol, 2014. 10(10): p. 2700-21.
	[12] Daraee, H., et al., Application of gold nanoparticles in biomedical and drug delivery. Artif Cells Nanomed Biotechnol,

	2016. 44(1): p. 410-22.
	[13] Kafshdooz, L., et al., The application of gold nanoparticles as a promising therapeutic approach in breast and ovarian cancer. Artif Cells Nanomed Biotechnol, 2016. 44(5): p. 1222-7.
	[14] Elahi, N., M. Kamali, and M.H. Baghersad, Recent biomedical applications of gold nanoparticles: A review. Talanta, 2018. 184: p. 537-556.
	[15] Liu, Y., B.M. Crawford, and T. Vo-Dinh, Gold nanoparticles-mediated photothermal therapy and immunotherapy. Immunotherapy, 2018. 10(13): p. 1175-1188.
	[16] Singh, P., et al., Gold Nanoparticles in Diagnostics and Therapeutics for Human Cancer. Int J Mol Sci, 2018. 19(7).
	[17] Aminabad, N.S., M. Farshbaf, and A. Akbarzadeh, Recent Advances of Gold Nanoparticles in Biomedical Applications: State of the Art. Cell Biochem Biophys, 2019. 77(2): p. 123-137.
	[18] Taghizadeh, S., et al., Gold nanoparticles application in liver cancer. Photodiagnosis Photodyn Ther, 2019. 25: p. 389-400.
	[19] Fuller, M.A. and I. Koper, Biomedical applications of polyelectrolyte coated spherical gold nanoparticles. Nano Converg, 2019. 6(1): p. 11.
**Biodistribution**	[20] Guo, J., et al., Gold nanoparticles enlighten the future of cancer theranostics. Int J Nanomedicine, 2017. 12: p. 6131-6152.
	[21] Hwang, S., et al., Gold nanoparticle-mediated photothermal therapy: current status and future perspective. Nanomedicine (Lond), 2014. 9(13): p. 2003-22.
	[22] Almeida, J.P., E.R. Figueroa, and R.A. Drezek, Gold nanoparticle mediated cancer immunotherapy. Nanomedicine, 2014. 10(3): p. 503-14.
**Catalytic**	[23] Song, S., et al., Functional nanoprobes for ultrasensitive detection of biomolecules. Chem Soc Rev, 2010. 39(11): p. 4234-43.
	[24] Jans, H. and Q. Huo, Gold nanoparticle-enabled biological and chemical detection and analysis. Chem Soc Rev, 2012. 41(7): p. 2849-66.

	[25] Wang, J. and X. Qu, Recent progress in nanosensors for sensitive detection of biomolecules. Nanoscale, 2013. 5(9): p. 3589-600.
	[11] Nie, L., et al., Applications of gold nanoparticles in optical biosensors. J Biomed Nanotechnol, 2014. 10(10): p. 2700-21.
	[26] Lo, V.K., A.O. Chan, and C.M. Che, Gold and silver catalysis: from organic transformation to bioconjugation. Org Biomol Chem, 2015. 13(24): p. 6667-80.
	[27] Jiang, P., et al., Applications of Gold Nanoparticles in Non-Optical Biosensors. Nanomaterials (Basel), 2018. 8(12).
	[28] Chang, C.C., et al., Gold Nanoparticle-Based Colorimetric Strategies for Chemical and Biological Sensing Applications. Nanomaterials (Basel), 2019. 9(6).
**Colloidal stability**	[29] Falahati, M., et al., Gold nanomaterials as key suppliers in biological and chemical sensing, catalysis, and medicine. Biochim Biophys Acta Gen Subj, 2020. 1864(1): p. 129435.
**Drug delivery**	[30] Kharisov, B.I., O.V. Kharissova, and S.S. Berdonosov, Radioactive nanoparticles and their main applications: recent advances. Recent Pat Nanotechnol, 2014. 8(2): p. 79-96.
	[12] Daraee, H., et al., Application of gold nanoparticles in biomedical and drug delivery. Artif Cells Nanomed Biotechnol, 2016. 44(1): p. 410-22.
	[14] Elahi, N., M. Kamali, and M.H. Baghersad, Recent biomedical applications of gold nanoparticles: A review. Talanta, 2018. 184: p. 537-556.
	[16] Singh, P., et al., Gold Nanoparticles in Diagnostics and Therapeutics for Human Cancer. Int J Mol Sci, 2018. 19(7).
	[31] Xu, J., et al., Research progress of novel inorganic nanometre materials carriers in nanomedicine for cancer diagnosis and treatment. Artif Cells Nanomed Biotechnol, 2018. 46(sup3): p. S492-S502.
	[17] Aminabad, N.S., M. Farshbaf, and A. Akbarzadeh, Recent Advances of Gold Nanoparticles in Biomedical Applications: State of the Art. Cell Biochem Biophys, 2019. 77(2): p. 123-137.
	[32] Das, P., et al., Multifunctional Magnetic Gold Nanomaterials

	for Cancer. Trends Biotechnol, 2019. 37(9): p. 995-1010.
	[19] Fuller, M.A. and I. Koper, Biomedical applications of polyelectrolyte coated spherical gold nanoparticles. Nano Converg, 2019. 6(1): p. 11.
	[33] Masse, F., et al., Gold nanoparticles in ophthalmology. Med Res Rev, 2019. 39(1): p. 302-327.
	[34] Mioc, A., et al., Gold Nanoparticles as Targeted Delivery Systems and Theranostic Agents in Cancer Therapy. Curr Med Chem, 2019. 26(35): p. 6493-6513.
	[35] Sharifi, M., et al., Plasmonic gold nanoparticles: Optical manipulation, imaging, drug delivery and therapy. J Control Release, 2019. 311-312: p. 170-189.
	[36] Singh, R., Nanotechnology based therapeutic application in cancer diagnosis and therapy. 3 Biotech, 2019. 9(11): p. 415.
**Localized surface plasmon resonance**	[11] Nie, L., et al., Applications of gold nanoparticles in optical biosensors. J Biomed Nanotechnol, 2014. 10(10): p. 2700-21.
	[37] Samanta, A., S. Banerjee, and Y. Liu, DNA nanotechnology for nanophotonic applications. Nanoscale, 2015. 7(6): p. 2210-20.
	[38] Ahmad, R., et al., Advanced Gold Nanomaterials for Photothermal Therapy of Cancer. J Nanosci Nanotechnol, 2016. 16(1): p. 67-80.
	[39] Kim, H., et al., Near-infrared light-responsive nanomaterials for cancer theranostics. Wiley Interdiscip Rev Nanomed Nanobiotechnol, 2016. 8(1): p. 23-45.
	[20] Guo, J., et al., Gold nanoparticles enlighten the future of cancer theranostics. Int J Nanomedicine, 2017. 12: p. 6131-6152.
	[14] Elahi, N., M. Kamali, and M.H. Baghersad, Recent biomedical applications of gold nanoparticles: A review. Talanta, 2018. 184: p. 537-556.
	[40] Hussein, E.A., et al., Recent advances in functional nanostructures as cancer photothermal therapy. Int J Nanomedicine, 2018. 13: p. 2897-2906.
	[15] Liu, Y., B.M. Crawford, and T. Vo-Dinh, Gold nanoparticles-mediated photothermal therapy and immunotherapy.

Immunotherapy, 2018. 10(13): p. 1175-1188.

[16] Singh, P., et al., Gold Nanoparticles in Diagnostics and Therapeutics for Human Cancer. Int J Mol Sci, 2018. 19(7).

[31] Xu, J., et al., Research progress of novel inorganic nanometre materials carriers in nanomedicine for cancer diagnosis and treatment. Artif Cells Nanomed Biotechnol, 2018. 46(sup3): p. S492-S502.

[41] Yadav, P., et al., Gold laced bio-macromolecules for theranostic application. Int J Biol Macromol, 2018. 110: p. 39-53.

[42] Ashikbayeva, Z., et al., Application of Nanoparticles and Nanomaterials in Thermal Ablation Therapy of Cancer. Nanomaterials (Basel), 2019. 9(9).

[28] Chang, C.C., et al., Gold Nanoparticle-Based Colorimetric Strategies for Chemical and Biological Sensing Applications. Nanomaterials (Basel), 2019. 9(6).

[32] Das, P., et al., Multifunctional Magnetic Gold Nanomaterials for Cancer. Trends Biotechnol, 2019. 37(9): p. 995-1010.

[19] Fuller, M.A. and I. Koper, Biomedical applications of polyelectrolyte coated spherical gold nanoparticles. Nano Converg, 2019. 6(1): p. 11.

[33] Masse, F., et al., Gold nanoparticles in ophthalmology. Med Res Rev, 2019. 39(1): p. 302-327.

[34] Mioc, A., et al., Gold Nanoparticles as Targeted Delivery Systems and Theranostic Agents in Cancer Therapy. Curr Med Chem, 2019. 26(35): p. 6493-6513.

[35] Sharifi, M., et al., Plasmonic gold nanoparticles: Optical manipulation, imaging, drug delivery and therapy. J Control Release, 2019. 311-312: p. 170-189.

[36] Singh, R., Nanotechnology based therapeutic application in cancer diagnosis and therapy. 3 Biotech, 2019. 9(11): p. 415.

[43] Son, J., et al., Light-responsive nanomedicine for biophotonic imaging and targeted therapy. Adv Drug Deliv Rev, 2019. 138: p. 133-147.

[44] Vines, J.B., et al., Gold Nanoparticles for Photothermal

	Cancer Therapy. Front Chem, 2019. 7: p. 167.
	[45] Khlebtsov, N.G., et al., Gap-enhanced Raman tags: fabrication, optical properties, and theranostic applications. Theranostics, 2020. 10(5): p. 2067-2094.
**Photochemistry**	[46] Urban, C., et al., Externally modulated theranostic nanoparticles. Transl Cancer Res, 2013. 2(4): p. 292-308.
**Photophysics**	[47] Susie, E. and e.-S.M. A, Why gold nanoparticles are more precious than pretty gold: noble metal surface plasmon resonance and its enhancement of the radiative and nonradiative properties of nanocrystals of different shapes. Chemical Society reviews, 2006. 35(3).
**Radioactivity**	[6] Coulter, J.A., et al., Radiosensitising nanoparticles as novel cancer therapeutics--pipe dream or realistic prospect? Clin Oncol (R Coll Radiol), 2013. 25(10): p. 593-603.
	[30] Kharisov, B.I., O.V. Kharissova, and S.S. Berdonosov, Radioactive nanoparticles and their main applications: recent advances. Recent Pat Nanotechnol, 2014. 8(2): p. 79-96.
**Self-assembly**	[48] Wu, C.S., F.K. Liu, and F.H. Ko, Potential role of gold nanoparticles for improved analytical methods: an introduction to characterizations and applications. Anal Bioanal Chem, 2011. 399(1): p. 103-18.
	[49] He, X.P., et al., Probing disease-related proteins with fluorogenic composite materials. Chem Soc Rev, 2015. 44(13): p. 4239-4248.
	[37] Samanta, A., S. Banerjee, and Y. Liu, DNA nanotechnology for nanophotonic applications. Nanoscale, 2015. 7(6): p. 2210-20.
	[50] Song, J., G. Niu, and X. Chen, Amphiphilic-Polymer-Guided Plasmonic Assemblies and Their Biomedical Applications. Bioconjug Chem, 2017. 28(1): p. 105-114.
	[31] Xu, J., et al., Research progress of novel inorganic nanometre materials carriers in nanomedicine for cancer diagnosis and treatment. Artif Cells Nanomed Biotechnol, 2018. 46(sup3): p. S492-S502.
**Toxicity**	[51] Gerber, A., et al., Gold nanoparticles: recent aspects for human toxicology. J Occup Med Toxicol, 2013. 8(1): p. 32.

	[52] Li, J., et al., Toxicity of inorganic nanomaterials in biomedical imaging. Biotechnol Adv, 2014. 32(4): p. 727-43.
	[53] Hadrup, N., et al., Toxicological risk assessment of elemental gold following oral exposure to sheets and nanoparticles - A review. Regul Toxicol Pharmacol, 2015. 72(2): p. 216-21.
	[54] De Matteis, V., Exposure to Inorganic Nanoparticles: Routes of Entry, Immune Response, Biodistribution and In Vitro/In Vivo Toxicity Evaluation. Toxics, 2017. 5(4).
	[55] Y.-P. Jia, B.-Y. Ma, X.-W. Wei, Z.-Y. Qian, The in vitro and in vivo toxicity of gold nanoparticles, Chinese Chemical Letters 28(04) (2017) 691-702.
	[16] Singh, P., et al., Gold Nanoparticles in Diagnostics and Therapeutics for Human Cancer. Int J Mol Sci, 2018. 19(7).

1. Hao, E., G.C. Schatz, and J.T. Hupp, *Synthesis and optical properties of anisotropic metal nanoparticles.* J Fluoresc, 2004. **14**(4): p. 331-41.
2. Li, N., P. Zhao, and D. Astruc, *Anisotropic gold nanoparticles: synthesis, properties, applications, and toxicity.* Angew Chem Int Ed Engl, 2014. **53**(7): p. 1756-89.
3. Jones, S., et al., *Recent progress on the development of anisotropic gold nanoparticles: Design strategies and growth mechanism.* J Environ Sci Health C Environ Carcinog Ecotoxicol Rev, 2017. **35**(1): p. 47-66.
4. Reguera, J., et al., *Anisotropic metal nanoparticles for surface enhanced Raman scattering.* Chem Soc Rev, 2017. **46**(13): p. 3866-3885.
5. Kohout, C., C. Santi, and L. Polito, *Anisotropic Gold Nanoparticles in Biomedical Applications.* Int J Mol Sci, 2018. **19**(11).
6. Coulter, J.A., et al., *Radiosensitising nanoparticles as novel cancer therapeutics--pipe dream or realistic prospect?* Clin Oncol (R Coll Radiol), 2013. **25**(10): p. 593-603.
7. Popescu, R.C., M.O. Fufa, and A.M. Grumezescu, *Metal-based nanosystems for diagnosis.* Rom J Morphol Embryol, 2015. **56**(2 Suppl): p. 635-49.
8. Torrisi, L., *Gold nanoparticles enhancing protontherapy efficiency.* Recent Pat Nanotechnol, 2015. **9**(1): p. 51-60.
9. Titus, D., E.J. Samuel, and S. Mohana Roopan, *Current scenario of biomedical aspect of metal-based nanoparticles on gel dosimetry.* Appl Microbiol Biotechnol, 2016. **100**(11): p. 4803-16.
10. Azharuddin, M., et al., *A repertoire of biomedical applications of noble metal nanoparticles.*

Chem Commun (Camb), 2019. **55**(49): p. 6964-6996.

11. Nie, L., et al., *Applications of gold nanoparticles in optical biosensors.* J Biomed Nanotechnol, 2014. **10**(10): p. 2700-21.

12. Daraee, H., et al., *Application of gold nanoparticles in biomedical and drug delivery.* Artif Cells Nanomed Biotechnol, 2016. **44**(1): p. 410-22.

13. Kafshdooz, L., et al., *The application of gold nanoparticles as a promising therapeutic approach in breast and ovarian cancer.* Artif Cells Nanomed Biotechnol, 2016. **44**(5): p. 1222-7.

14. Elahi, N., M. Kamali, and M.H. Baghersad, *Recent biomedical applications of gold nanoparticles: A review.* Talanta, 2018. **184**: p. 537-556.

15. Liu, Y., B.M. Crawford, and T. Vo-Dinh, *Gold nanoparticles-mediated photothermal therapy and immunotherapy.* Immunotherapy, 2018. **10**(13): p. 1175-1188.

16. Singh, P., et al., *Gold Nanoparticles in Diagnostics and Therapeutics for Human Cancer.* Int J Mol Sci, 2018. **19**(7).

17. Aminabad, N.S., M. Farshbaf, and A. Akbarzadeh, *Recent Advances of Gold Nanoparticles in Biomedical Applications: State of the Art.* Cell Biochem Biophys, 2019. **77**(2): p. 123-137.

18. Taghizadeh, S., et al., *Gold nanoparticles application in liver cancer.* Photodiagnosis Photodyn Ther, 2019. **25**: p. 389-400.

19. Fuller, M.A. and I. Koper, *Biomedical applications of polyelectrolyte coated spherical gold nanoparticles.* Nano Converg, 2019. **6**(1): p. 11.

20. Guo, J., et al., *Gold nanoparticles enlighten the future of cancer theranostics.* Int J Nanomedicine, 2017. **12**: p. 6131-6152.

21. Hwang, S., et al., *Gold nanoparticle-mediated photothermal therapy: current status and future perspective.* Nanomedicine (Lond), 2014. **9**(13): p. 2003-22.

22. Almeida, J.P., E.R. Figueroa, and R.A. Drezek, *Gold nanoparticle mediated cancer immunotherapy.* Nanomedicine, 2014. **10**(3): p. 503-14.

23. Song, S., et al., *Functional nanoprobes for ultrasensitive detection of biomolecules.* Chem Soc Rev, 2010. **39**(11): p. 4234-43.

24. Jans, H. and Q. Huo, *Gold nanoparticle-enabled biological and chemical detection and analysis.* Chem Soc Rev, 2012. **41**(7): p. 2849-66.

25. Wang, J. and X. Qu, *Recent progress in nanosensors for sensitive detection of biomolecules.* Nanoscale, 2013. **5**(9): p. 3589-600.

26. Lo, V.K., A.O. Chan, and C.M. Che, *Gold and silver catalysis: from organic transformation to bioconjugation.* Org Biomol Chem, 2015. **13**(24): p. 6667-80.

27. Jiang, P., et al., *Applications of Gold Nanoparticles in Non-Optical Biosensors.* Nanomaterials (Basel), 2018. **8**(12).

28. Chang, C.C., et al., *Gold Nanoparticle-Based Colorimetric Strategies for Chemical and Biological Sensing Applications.* Nanomaterials (Basel), 2019. **9**(6).

29. Falahati, M., et al., *Gold nanomaterials as key suppliers in biological and chemical sensing, catalysis, and medicine.* Biochim Biophys Acta Gen Subj, 2020. **1864**(1): p. 129435.

30. Kharisov, B.I., O.V. Kharissova, and S.S. Berdonosov, *Radioactive nanoparticles and their main applications: recent advances.* Recent Pat Nanotechnol, 2014. **8**(2): p. 79-96.

31. Xu, J., et al., *Research progress of novel inorganic nanometre materials carriers in nanomedicine for cancer diagnosis and treatment.* Artif Cells Nanomed Biotechnol, 2018. **46**(sup3): p. S492-S502.

32. Das, P., et al., *Multifunctional Magnetic Gold Nanomaterials for Cancer.* Trends Biotechnol, 2019. **37**(9): p. 995-1010.

33. Masse, F., et al., *Gold nanoparticles in ophthalmology.* Med Res Rev, 2019. **39**(1): p. 302-327.

34. Mioc, A., et al., *Gold Nanoparticles as Targeted Delivery Systems and Theranostic Agents in Cancer Therapy.* Curr Med Chem, 2019. **26**(35): p. 6493-6513.

35. Sharifi, M., et al., *Plasmonic gold nanoparticles: Optical manipulation, imaging, drug delivery and therapy.* J Control Release, 2019. **311-312**: p. 170-189.

36. Singh, R., *Nanotechnology based therapeutic application in cancer diagnosis and therapy.* 3 Biotech, 2019. **9**(11): p. 415.

37. Samanta, A., S. Banerjee, and Y. Liu, *DNA nanotechnology for nanophotonic applications.* Nanoscale, 2015. **7**(6): p. 2210-20.

38. Ahmad, R., et al., *Advanced Gold Nanomaterials for Photothermal Therapy of Cancer.* J Nanosci Nanotechnol, 2016. **16**(1): p. 67-80.

39. Kim, H., et al., *Near-infrared light-responsive nanomaterials for cancer theranostics.* Wiley Interdiscip Rev Nanomed Nanobiotechnol, 2016. **8**(1): p. 23-45.

40. Hussein, E.A., et al., *Recent advances in functional nanostructures as cancer photothermal therapy.* Int J Nanomedicine, 2018. **13**: p. 2897-2906.

41. Yadav, P., et al., *Gold laced bio-macromolecules for theranostic application.* Int J Biol Macromol, 2018. **110**: p. 39-53.

42. Ashikbayeva, Z., et al., *Application of Nanoparticles and Nanomaterials in Thermal Ablation Therapy of Cancer.* Nanomaterials (Basel), 2019. **9**(9).

43. Son, J., et al., *Light-responsive nanomedicine for biophotonic imaging and targeted therapy.*

Adv Drug Deliv Rev, 2019. **138**: p. 133-147.

44. Vines, J.B., et al., *Gold Nanoparticles for Photothermal Cancer Therapy.* Front Chem, 2019. **7**: p. 167.

45. Khlebtsov, N.G., et al., *Gap-enhanced Raman tags: fabrication, optical properties, and theranostic applications.* Theranostics, 2020. **10**(5): p. 2067-2094.

46. Urban, C., et al., *Externally modulated theranostic nanoparticles.* Transl Cancer Res, 2013. **2**(4): p. 292-308.

47. Susie, E. and e.-S.M. A, *Why gold nanoparticles are more precious than pretty gold: noble metal surface plasmon resonance and its enhancement of the radiative and nonradiative properties of nanocrystals of different shapes.* Chemical Society reviews, 2006. **35**(3).

48. Wu, C.S., F.K. Liu, and F.H. Ko, *Potential role of gold nanoparticles for improved analytical methods: an introduction to characterizations and applications.* Anal Bioanal Chem, 2011. **399**(1): p. 103-18.

49. He, X.P., et al., *Probing disease-related proteins with fluorogenic composite materials.* Chem Soc Rev, 2015. **44**(13): p. 4239-4248.

50. Song, J., G. Niu, and X. Chen, *Amphiphilic-Polymer-Guided Plasmonic Assemblies and Their Biomedical Applications.* Bioconjug Chem, 2017. **28**(1): p. 105-114.

51. Gerber, A., et al., *Gold nanoparticles: recent aspects for human toxicology.* J Occup Med Toxicol, 2013. **8**(1): p. 32.

52. Li, J., et al., *Toxicity of inorganic nanomaterials in biomedical imaging.* Biotechnol Adv, 2014. **32**(4): p. 727-43.

53. Hadrup, N., et al., *Toxicological risk assessment of elemental gold following oral exposure to sheets and nanoparticles - A review.* Regul Toxicol Pharmacol, 2015. **72**(2): p. 216-21.

54. De Matteis, V., *Exposure to Inorganic Nanoparticles: Routes of Entry, Immune Response, Biodistribution and In Vitro/In Vivo Toxicity Evaluation.* Toxics, 2017. **5**(4).

55. Jia, Y.-P., et al., *The in vitro and in vivo toxicity of gold nanoparticles.* Chinese Chemical Letters, 2017. **28**(04): p. 691-702.
